# The Relationship between Conscientiousness and Well-Being among Chinese Undergraduate Students: A Cross-Lagged Study

**DOI:** 10.3390/ijerph192013565

**Published:** 2022-10-19

**Authors:** Yaqi Hu, Zhenhong Wang, Qing Fan

**Affiliations:** Shaanxi Provincial Key Research Center of Child Mental and Behavioral Health, Shaanxi Key Laboratory of Behavior and Cognitive Neuroscience, School of Psychology, Shaanxi Normal University, Xi’an 710062, China

**Keywords:** conscientiousness, physical well-being, subjective well-being

## Abstract

Chinese culture attaches great importance to the education and cultivation of youth conscientiousness, however in the context of Chinese culture, little is known about the relationship between conscientiousness and mental and physical health. The present study aimed to investigate whether there is a reciprocal relationship between conscientiousness and well-being (subjective and physical well-being) among Chinese undergraduate students. A series of self-reported questionnaires were administered to 365 undergraduate students in 2 waves, separated by 1 year. Cross-lagged regression analyses were applied to examine the reciprocal relationships. Results indicated that conscientiousness positively predicted subsequent levels of positive affect and life satisfaction, while negatively predicted subsequent levels of negative affect and physical symptoms, controlling for the effects of gender, age, body-mass index, socioeconomic status, and the prior level of conscientiousness. Whereas, positive and negative affect, life satisfaction, and physical symptoms did not significantly predict subsequent levels of conscientiousness. This study suggests that conscientiousness is a robust and prospective predictor of subjective and physical well-being. The reciprocal relationship between conscientiousness and well-being was not confirmed in the current sample of Chinese undergraduate students.

## 1. Introduction

Over the past few decades, a great number of studies have linked personality traits with well-being [1,2,3]. Among the Big Five personality traits, conscientiousness reflects a set of characteristics describing industriousness, orderliness, goal-directed, planning, impulse control, delayed gratification, active coping, and the propensity to adhere to societal rules and norms [4], which is related to a serial of social adaptive outcomes, such as better work performance [5], higher career self-efficacy [6], positive interpersonal relationships [7] and higher academic effort [8] or achievement [9]. Notably, conscientiousness was found to be closely linked with an individual’s mental and physical health [10]. Some studies have also proposed and preliminarily found a reciprocal relationship between conscientiousness and subjective and physical well-being [11,12]. However, existing studies provide only limited evidence on the reciprocal relationship. The present study sought to test whether there is a reciprocal relationship between conscientiousness and subjective and physical well-being among Chinese undergraduate students.

### 1.1. Relation between Conscientiousness and Subjective Well-Being

Subjective well-being refers to the personal evaluations of positive affect, negative affect, and life satisfaction [13]. A large number of cross-sectional studies have shown that conscientious individuals tend to experience more frequent positive affect, greater life satisfaction, and less frequent negative affect as compared to less conscientious individuals [14,15,16,17]. Individuals high in conscientiousness are more effective at regulating negative affect [18], and are less likely to suffer from depression and anxiety disorders [19]. Recently, a longitudinal study observed that individuals who were initially conscientious subsequently report increased subjective well-being, and those with high initial levels of subjective well-being subsequently become more conscientious [12]. In addition, a longitudinal study from Specht et al. [20] suggested that life satisfaction prospectively predicted improvements in conscientiousness. These research findings indicate that there is a reciprocal relationship between conscientiousness and subjective well-being.

### 1.2. Relation between Conscientiousness and Physical Well-Being

Moreover, studies have investigated the relationship between conscientiousness and physical well-being, conceptualized as the absence of illness or physical symptoms, and maintaining normal physical functioning [3,21]. Conscientiousness was observed to be negatively associated with inflammation [22] and risks of chronic illness, such as sciatica, stroke, hypertension, and diabetes [23,24,25]. Accumulating evidence has consistently suggested that conscientiousness is a protective factor against physical health problems [26,27,28]. Recent findings from empirical studies have shown that individuals high in conscientiousness tend to report better physical health status [10,29,30]. A small number of longitudinal studies on the influence of conscientiousness on physical well-being have suggested that an increase in conscientiousness predicts improved self-reported health [31,32,33,34]. Roberts et al. [11] proposed that, similar to subjective well-being, there is a reciprocal relationship between conscientiousness and physical health. That is, favorable health behaviors and life paths that contribute to physical health resulting partially from being conscientious, may conversely facilitate increased conscientiousness. However, few empirical studies have examined the influence of physical well-being on conscientiousness, and thus, the reciprocal relationship between conscientiousness and physical well-being remains unclear.

### 1.3. Cultural Influences

Examining relationships between conscientiousness and subjective and physical well-being in the context of Chinese culture, which attaches great importance to the cultivation of youth conscientiousness, can help to provide us with a more in-depth understanding of the relationship between conscientiousness and mental and physical health. Cultural differences in the links between personality traits and health-related outcomes can be explained by differences in behaviors and lifestyles [35], in other words, there are multiple pathways to achieve or maintain happiness and health, and they are somewhat different across the internalized cultural values, which have permeated and influenced individuals’ subjective well-being [36] and health-seeking decisions [37]. For example, recent research has shown that conscientiousness is likely to have a stronger protective effect on stress perception in an eastern culture than in a western culture [38]. Additionally, the core factor in the process of personality development is the individual’s self-concepts formed in a certain cultural environment [39]. Chinese youth acquire values, norms, and beliefs from traditional cultures that emphasize individual obligations and the needs of others [40]. 

### 1.4. The Present Study

Taken together, extant cross-sectional studies have demonstrated that conscientiousness is a robust predictor of subjective and physical well-being. Some longitudinal studies also revealed a reciprocal relationship between conscientiousness and subjective well-being. In addition, some researchers believe there is a reciprocal relationship between conscientiousness and physical health [11,12]. Given the limited research available, reciprocal relationships between conscientiousness and subjective and physical well-being need to be further examined. Particularly in the Chinese culture, the relationships between conscientiousness and aspects of well-being have received scant attention from investigators, even though conscientiousness is a highly valued personality trait. Therefore, the present study adopted a cross-lagged design to examine the reciprocal influence pattern of conscientiousness with subjective well-being (indexed by positive affect, negative affect, and life satisfaction) and physical well-being (indexed by self-reported physical symptoms) among Chinese undergraduate students. The cross-lagged design is useful in assessing reciprocal relationships by examining the asymmetry of the predicted association between each variable at one point and another variable at a later point in time [41]. Based on preceding research findings [11,12], we hypothesized that there would be reciprocal relationships between conscientiousness and subjective and physical well-being over time.

## 2. Methods

### 2.1. Participants and Procedure

Undergraduates from a university in northwest China were recruited by posting flyers around the campus and online. All participants were informed that the purpose of this longitudinal study was to investigate undergraduate students’ subjective and physical well-being via questionnaires. They reported voluntary participation and provided informed consent prior to the study, following which they were asked to complete a series of questionnaires in 2 waves during the period of the investigation. A total of 420 valid questionnaires were collected in the first wave (at T1: 16 September 2018 to 28 September 2018) from 282 females and 138 males. The mean age of the sample was 18.98 years (*SD* = 0.96) ranging from 17 to 25 years. After 1 year (at T2: 13 September 2019 to 27 September 2019), follow-up telephone or e-mail invitations were sent to the 420 participants inviting them to participate in the second survey wave. Data from 55 participants were excluded due to incomplete responses, academic interruption, internship search, or other matters. Ultimately, a total of 365 valid respondents (*M*_age_ = 19.95 years, *SD* = 0.93, range = 18–26 years old; 67.95% females) participated in the two waves. There were no age differences between males and females, *t*_(363)_ = 0.174, *p* > 0.05, Cohen’s *d* = 0.02. The participants who did not participate in the second wave were not significantly different from those who participated in the two waves in terms of any T1 measures (for age and the main study variables: all *t* values were less than 1.31, *p*s > 0.05; for gender and other ordinal demographic characteristics, all *χ*^2^ values were less than 6.44, *p*s > 0.05).

### 2.2. Measures

#### 2.2.1. Demographic and Socioeconomic Background

Two waves of demographic data were collected, including gender, age, body-mass index (BMI), household monthly income, and parents’ education levels. BMI was calculated from weight and height (kg/m^2^). Household monthly income was categorized into four levels: (1) *<3000 RMB*; (2) *3000–7000 RMB*; (3) *7000–10,000 RMB*; and (4) >*10,000 RMB*. Parents’ educational levels were coded as 1 = *never attended any school*, 2 = *primary school*, 3 = *junior high school*, 4 = *high school*, 5 = *junior college or undergraduate*, and 6 = *graduate* (*master or doctor*). According to recommendations in the literature [42], socioeconomic status (SES) scores were calculated by summing standardized scores of the household monthly income level and the parents’ education level, whereby high scores indicated high SES. Gender, age, BMI, and SES were utilized as control variables in the analyses described below.

#### 2.2.2. Conscientiousness

Conscientiousness was assessed using a 12-item scale selected from the shortened Chinese version of the NEO Five-Factor Inventory (NEO-FFI) [43]. All items (e.g., “I try to be the best at anything I do”) were rated on a 5-point Likert scale, ranging from 1 (*strongly disagree*) to 5 (*strongly agree*). This scale showed good reliability and validity in the Chinese sample [44]. In the present study, Cronbach’s alphas for conscientiousness were 0.84 at T1 and 0.87 at T2.

#### 2.2.3. Positive and Negative Affect

Emotional well-being was evaluated using the Positive and Negative Affect Schedule (PANAS) [45], which is a self-rated measure of positive affect (PA) and negative affect (NA). Huang et al. [46] have verified that the PANAS is appropriate for use with Chinese populations, and shows good reliability and validity. The scale consists of 10 affective adjectives for PA (e.g., “attentive”, “excited”, and “inspired”) and 10 affective adjectives for NA (e.g., “ashamed”, “guilty”, and “irritable”). Each participant rated how they felt in general from 1 (*very slightly or not at all*) to 5 (*extremely*). Cronbach’s alpha coefficients were 0.85 for PA at T1, 0.84 for NA at T1, 0.86 for PA at T2, and 0.87 for NA at T2.

#### 2.2.4. Life Satisfaction

The Satisfaction with Life Scale (SWLS) [47] is widely used to assess the cognitive component of subjective well-being. The standardized Chinese version of the SWLS was adopted in the present study [48]. Respondents were asked to indicate the degree to which each item was true for them on the 5-item scale (e.g., “in most ways, my life is close to ideal”) from 1 (*disagree strongly*) to 7 (*agree strongly*). Cronbach’s alphas for the scale were 0.90 at T1 and 0.89 at T2.

#### 2.2.5. Physical Symptoms

The Cohen–Hoberman Inventory of Physical Symptoms (CHIPS) [49,50] was designed to assess general physical symptoms. The Chinese version of the CHIPS was proved to have good reliability and validity in the Chinese sample [51]. This scale comprises a list of 33 common symptoms (e.g., “acne”, “back pain”, and “heart pounding or racing”). Respondents were required to rate “how much that problem has bothered or distressed you during the past 2 weeks?” on a 5-point Likert scale ranging from 0 (*not been bothered by the problem*) to 4 (*extremely bothered by the problem*), with higher total scores indicating lower levels of physical well-being. Cronbach’s alphas in this study were good in the two waves (T1: α = 0.94; T2: α = 0.93).

### 2.3. Data Analytical Strategies

Descriptive analysis and partial correlation analysis were conducted on the key variables at T1 and T2. Paired *t* tests were then performed to detect differences in these key variables between the two waves. Finally, a set of cross-lagged regression analyses were adopted to assess the reciprocal relationship between conscientiousness and well-being. All statistical analyses were conducted using IBM SPSS Statistics 25.0.

### 2.4. Assessment of Common Method Biases

Since all study variables utilized self-rating measures, a Harman single factor test was employed to assess the common method bias [52]. A total of 70 items from the questionnaires at T1 were subjected to the exploratory factor analysis without rotation. The analysis produced 17 common factors, with the first explaining the 19.17% variance, and the analysis of 70 items at T2 yielded 17 factors, with the first explaining the 19.40% variance, both of which were lower than the critical standard of 40% [53]. The results indicated that the correlation between the studied variables in this study was not driven purely by method bias.

## 3. Results

### 3.1. Demographic Characteristics

The distribution of all sociodemographic variables at T1 is shown in Table 1. On average, participants were 19 years of age, and approximately 67.95% were females. Almost 93.42% of the participants reported that their family monthly income was less than 10,000 RMB (approximately $1426). With respect to parents’ education levels, 83.29% of the fathers and 72.33% of the mothers had at least a junior high school degree. In addition, 1.92% of the fathers and 0.55% of the mothers were either masters or doctorates.

### 3.2. Descriptive and Bivariate Analyses

Descriptive statistics for the main variables at T1 and T2 are presented in Table 2. Next, a partial correlation analysis was performed with gender, age, BMI, and SES as control variables. As expected, conscientiousness, positive affect, negative affect, life satisfaction, and physical symptoms at T1 were significantly correlated with those at T2 (*r* = 0.65, *p* < 0.001; *r* = 0.50, *p* < 0.001; *r* = 0.46, *p* < 0.001; *r* = 0.55, *p* < 0.001; *r* = 0.51, *p* < 0.001), indicating a certain degree of temporal stability separated by 1 year.

T1 conscientiousness was significantly correlated with positive affect (*r* = 0.37, *p* < 0.001; *r* = 0.33, *p* < 0.001), negative affect (*r* = −0.21, *p* < 0.001; *r* = −0.21, *p* < 0.001), life satisfaction (*r* = 0.34, *p* < 0.001; *r* = 0.28, *p* < 0.001), and physical symptoms (*r* = −0.23, *p* < 0.001; *r* = −0.22, *p* < 0.001) at T1 and T2. Additionally, T2 conscientiousness was significantly correlated with positive affect (*r* = 0.26, *p* < 0.001; *r* = 0.27, *p* < 0.001), negative affect (*r* = −0.14, *p* = 0.008; *r* = −0.27, *p* < 0.001), life satisfaction (*r* = 0.28, *p* < 0.001; *r* = 0.29, *p* < 0.001), and physical symptoms (*r* = −0.16, *p* = 0.002; *r* = −0.19, *p* < 0.001) at T1 and T2. These findings suggested that both simultaneous and sequential correlations between conscientiousness and well-being were significant, which satisfied the prior assumption of the cross-lagged panel correlation paradigm.

Further results from a set of paired *t* tests showed that: T2 positive affect was significantly lower than T1 positive affect, *t*_(364)_ = 2.76, *p* = 0.006, Cohen’s *d* = 0.14 (small effect size); T2 life satisfaction was significantly lower than T1 life satisfaction, *t*_(364)_ = 5.81, *p* < 0.001, Cohen’s *d* = 0.30 (small effect size); conscientiousness, negative affect and physical symptoms did not differ significantly in the two waves (*t*_(364)_ = 1.79, *p* = 0.075, Cohen’s *d* = 0.09; *t*_(364)_ = −0.643, *p* = 0.521, Cohen’s *d* = 0.03; *t*_(364)_ = 0.621, *p* = 0.535, Cohen’s *d* = 0.03).

### 3.3. Cross-Lagged Regression Analyses

A set of multivariate hierarchical regressions for cross-lagged panel analyses were conducted to examine the mutual predictive relationship between conscientiousness and each aspect of well-being across time. Specifically, variables were entered at 2 steps: (1) gender, age, BMI, SES, and the T1 measure of the outcome variable were entered simultaneously as control variables, and (2) T1 conscientiousness was entered as the predictor variable, along with T2 well-being as the outcome variable (Additional analysis: T1 well-being was entered as the predictor variable, along with T2 conscientiousness as the outcome variable). All continuous variables were standardized before entering the equation. Each regression included a collinearity diagnosis test, indicating no overlap among control variables and predictors (tolerance values were 0.83–0.99). The regression analysis results for T1 conscientiousness predicting subjective and physical well-being at T2 are shown in Table 3.

With T2 positive affect as the outcome variable, all predictors in the model accounted for 29.00% of the variance. In addition to the contribution of control variables (26.70%), the inclusion of T1 conscientiousness explained an additional 2.30% of the variance, Δ*F* (1, 358) = 11.53, *p* = 0.001. In the other models, likewise, the inclusion of T1 conscientiousness explained 1.40% of the variance in T2 negative affect (Δ*F* (1, 358) = 6.61, *p* = 0.011), 1.00% of the variance in T2 life satisfaction (Δ*F* (1, 358) = 5.49, *p* = 0.020), and 1.00% of the variance in T2 physical symptoms (Δ*F* (1, 358) = 5.01, *p* = 0.026), respectively. These findings suggested that after controlling for the effects of gender, age, BMI, SES and baseline values, T1 conscientiousness significantly predicted T2 positive affect, T2 negative affect, T2 life satisfaction, and T2 physical symptoms, respectively.

Additionally, the regression analysis results for T1 well-being predicting T2 conscientiousness are shown in Table 4. These findings suggested that the predictive effects of subjective and physical well-being at T1 on T2 conscientiousness were not statistically significant. In addition to the contribution of control variables, the contributions of subjective and physical well-being at T1 to the variance of T2 conscientiousness were not statistically significant, respectively (positive affect: Δ*R*^2^ = 0.001, Δ*F* (1, 358) = 0.48, *p* > 0.05; negative affect: Δ*R*^2^ = 0.000, Δ*F* (1, 358) = 0.02, *p* > 0.05; life satisfaction: Δ*R*^2^ = 0.004, Δ*F* (1, 358) = 2.28, *p* > 0.05; physical symptoms: Δ*R*^2^ = 0.000, Δ*F* (1, 358) = 0.03, *p* > 0.05).

**Figure 1:**
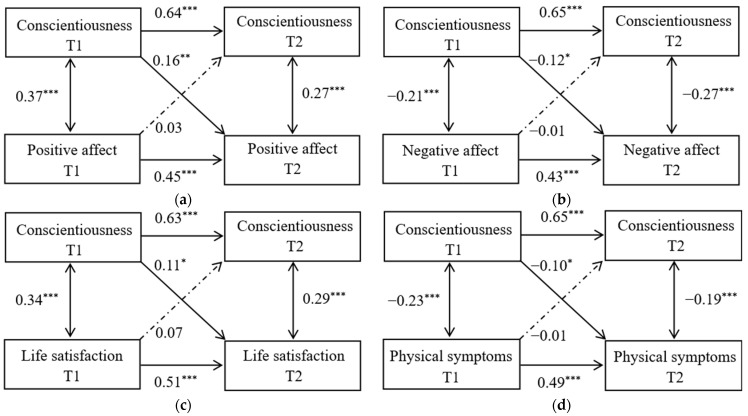
Multivariable cross-lagged panel models of (**a**) conscientiousness and positive affect, (**b**) conscientiousness and negative affect, (**c**) conscientiousness and life satisfaction, and (**d**) conscientiousness and physical symptoms. Note: The coefficients beside double arrows are partial correlations and the coefficients beside single arrows are standardized beta coefficients. Solid arrows represent significant paths and dotted arrows represent insignificant paths. The effects of demographic variables (gender, age, body-mass index [BMI], and socioeconomic status [SES]) on predicted variables were not shown for ease of presentation. T1: time 1, T2: time 2 (1 year later), * *p* < 0.05, ** *p* < 0.01, *** *p* < 0.001.

As depicted in Figure 1, conscientiousness prospectively predicted subjective and physical well-being, but subjective and physical well-being did not prospectively predict conscientiousness. Based on the recommendations of Orth et al. [54], 0.03 (small effect), 0.07 (medium effect), and 0.12 (large effect) can be used as benchmark values to interpret the size of cross-lagged effects. Thus, the cross-lagged effects (C-T1→PA-T2, C-T1→NA-T2, C-T1→LS-T2, C-T1→PS-T2) found in this study were statistically significant and above the medium effect size level. Consequently, it can be inferred that there may not be a reciprocal relationship between conscientiousness and subjective and physical well-being in the current sample.

## 4. Discussion

The present study aimed to extend research on the link between conscientiousness and health by examining whether there is a reciprocal relationship between conscientiousness and well-being (subjective and physical well-being) using a cross-lagged design in a sample of Chinese undergraduate students. The results indicated that after controlling for the effects of gender, age, BMI, SES, and the prior level of conscientiousness, conscientiousness prospectively predicted subjective well-being (indexed by positive affect, negative affect, and life satisfaction) and physical well-being (indexed by self-reported physical symptoms). However, subjective and physical well-being didn’t prospectively predict conscientiousness. Therefore, the reciprocal relationship between conscientiousness and well-being was not confirmed in the current sample of Chinese undergraduate students.

We found that conscientiousness prospectively predicted subjective well-being indexed by positive affect, negative affect, and life satisfaction among Chinese undergraduates. These findings replicated previous evidence that individuals with high conscientiousness reported more frequent positive affect, greater life satisfaction, and less frequent negative affect [12,14,16], revealing that conscientiousness is a robust and prospective predictor of subjective well-being. On the one hand, individuals high in conscientiousness are likely to be more effective at regulating negative affect [18] and may experience less daily stress [27,32]. On the other hand, individuals high in conscientiousness are more likely to be self-disciplined, goal-directed, industrious, and rule-abiding [4], and their efficient and reliable performance may promote success in school or the workplace [5,6,8,9]. Such success can help to enhance their subjective well-being [12]. Additionally, individuals high in conscientiousness may be more successful in building stable and satisfying interpersonal relationships, which in turn may contribute to enhanced feelings of subjective well-being. In contrast, individuals low in conscientiousness may have difficulty developing or maintaining adaptive interpersonal relationships, which in turn may contribute to decreased feelings of subjective well-being over time [7]. 

Similarly, our study found that conscientiousness prospectively predicted physical well-being indexed by self-reported physical symptoms. This finding is the preliminary evidence to demonstrate the predictive effect of conscientiousness on physical well-being in the sample of Chinese undergraduates, which is consistent with the results found in the samples from Western countries [10,29,55,56]. Individuals low in conscientiousness reported more physical symptoms and negative health outcomes as compared to individuals high in conscientiousness. Researchers have proposed that conscientiousness may protect physical health by promoting favorable health behaviors and avoiding detrimental health behaviors [11,57]. Conscientious individuals tend to pursue higher quality of life and healthier life paths, such as engaging in more physical exercise and outdoor activities, and adhering to treatment and medication because of being self-disciplined and responsible [58,59], which is more conducive to physical health. While less conscientious individuals tend to be characterized by poor self-control and self-discipline and often have unhealthy lifestyles, such as poor diet and lack of exercise, which increases the risk of chronic diseases [60,61,62]. Personality is a determining factor for key outcomes across the lifespan, with the similar predictive validity to socioeconomic status and cognitive ability [57]. Therefore, it is of great significance to identify conscientiousness as a personality indicator of physical well-being.

Contrary to our hypothesis, this preliminary exploration into the reciprocal relation between conscientiousness and well-being among Chinese undergraduate students did not find prospective predictive effects of subjective well-being and physical well-being on conscientiousness. In other words, individual differences in conscientiousness can explain differences in subjective and physical well-being, but are less likely to be explained by differences in subjective and physical well-being. In the context of Chinese culture, conscientiousness is a highly valued trait shaped by collectivist cultural values. School and family education in China especially pay attention to the cultivation of culturally valued traits among children and adolescents [63]. From childhood, students are guided to develop the right values, a proactive view of themselves, and culturally valued traits, especially conscientiousness. Individual personality traits do change after adolescence and most change for the better; the changes that occur in adulthood, while clearly for the better, are smaller in magnitude than during childhood and adolescence [64]. Thus, individual differences in conscientiousness are less likely to be affected by differences in well-being during emerging adulthood than during childhood and adolescence. At least in the sample of this study, we did not demonstrate that subjective and physical well-being were prospective predictors of conscientiousness over a one-year interval. Future research could explore the reciprocal relationships between conscientiousness and subjective and physical well-being in children and adolescents. 

### 4.1. Practical Implications

Not only can personality change, it can substantially change with the general maturation process and personal circumstances [64,65]. The findings of this study suggest that cultivating conscientiousness may help to improve physical and mental health, in addition to other interventions specifically designed to improve physical and mental well-being. Education policymakers and educators may wish to consider the following recommendations for interventions related to promoting conscientiousness, particularly for undergraduate students with low conscientiousness. First, create a safe, supportive, orderly, and minimally distracting environment that fosters students’ sense of belonging. Second, help students to internalize relevant social norms (e.g., rule compliance) to form internalized values. Third, cultivate developmentally appropriate skills related to conscientiousness, such as goal setting, time management, progress monitoring, and self-regulation. Finally, support students to work toward long-term goals that are achievable, challenging, and personally meaningful.

### 4.2. Limitations and Future Directions

Several limitations of the present study should be considered. First, the present study relied on a sample of undergraduates and thus the conclusions need to be further tested in a more general population in the future. Second, the present study used only a two-wave cross-lagged design with a one-year interval, a longitudinal design with more waves and longer time intervals should be adopted to establish the causal relationships between conscientiousness and well-being more reliably and accurately in future studies. Third, the lack of attention to balancing male and female participants in the study design limited the interpretation of the current findings, and future research should address this issue. Finally, this study included only self-reported physical symptoms as a measure of physical well-being. Future research could employ more objective indicators of physical well-being.

## 5. Conclusions

This study extended extant research by probing into whether there is a reciprocal relation between conscientiousness and well-being (subjective and physical well-being) using a two-wave cross-lagged design in a sample of Chinese undergraduate students. Our findings suggest that conscientiousness is a robust and prospective predictor of subjective and physical well-being, but subjective and physical well-being were not prospective predictors of conscientiousness. The present study contributes to an in-depth understanding of the conscientiousness-health relation. Promoting conscientiousness in future health-related interventions may help to foster better physical and mental health for undergraduate students, especially for those with low conscientiousness.

## Figures and Tables

**Table 1:** Sample characteristics for sociodemographic variables at Time 1 (*N* = 365).

	%/*M* (*SD*)	Range
Gender (% female)	67.95	
Age (in years)	18.95 (0.93)	17–25
BMI	20.89 (2.64)	15.80–32.40
SES	0.00 (2.39)	−4.73–6.92
Family monthly income		1–4
<3000 RMB	23.01	
3000–7000 RMB	51.78	
7000–10,000 RMB	18.63	
>10,000 RMB	6.58	
Father’s education level		1–6
never attended any school	0.82	
primary school	15.89	
junior high school	36.16	
high school	21.64	
junior college or undergraduate	23.56	
graduate (master or doctor)	1.92	
Mother’s education level		1–6
never attended any school	5.48	
primary school	22.19	
junior high school	34.79	
high school	18.63	
junior college or undergraduate	18.36	
graduate (master or doctor)	0.55	

Note. *M*: means; *SD*: standard deviations; BMI: body-mass index; SES: socioeconomic status.

**Table 2:** Means and standard deviations for the key variables (*N* = 365).

	T1 (Pre-Test)		T2 (Post-Test)	
	*M*	*SD*	*M*	*SD*
Conscientiousness	41.17	5.93	40.74	5.11
Positive Affect	29.43	6.08	28.58	5.90
Negative Affect	20.59	5.17	20.78	5.69
Life Satisfaction	21.08	5.44	19.45	5.98
Physical Symptoms	18.39	15.56	17.91	14.46

Note. *M*: means; *SD*: standard deviations.

**Table 3:** Separate regression models for T1 conscientiousness predicting subjective and physical well-being at T2.

Predictor	PA-T2				NA-T2			
	Model 1		Model 2		Model 1		Model 2	
	β	*t*	β	*t*	β	*t*	β	*t*
**Step 1**								
Gender	−0.06	−0.57	−0.08	−0.80	0.01	0.07	0.01	0.11
Age	0.02	0.47	0.02	0.32	−0.01	−0.13	0.01	0.14
BMI	0.01	0.27	0.02	0.47	0.07	1.33	0.06	1.27
SES	0.01	0.11	−0.01	−0.10	−0.06	−1.27	−0.05	−1.01
T1 measure	0.51	11.04 ***	0.45	9.19 ***	0.46	9.76 ***	0.43	9.10 ***
**Step 2**								
C-T1			0.16	3.40 **			−0.12	−2.57 *
	Total *R*^2^ = 0.29, *F* (6, 358) = 24.31 ***, Δ*R*^2^ = 0.023				Total *R*^2^ = 0.24, *F* (6, 358) = 18.93 ***, Δ*R*^2^ = 0.014			
	**LS-T2**				**PS-T2**			
	**Model 1**		**Model 2**		**Model 1**		**Model 2**	
	**β**	***t***	**β**	***t***	**β**	***t***	**β**	***t***
**Step 1**								
Gender	−0.05	−0.52	−0.05	−0.54	−0.03	−0.26	−0.02	−0.22
Age	0.02	0.50	0.02	0.32	−0.05	−1.04	−0.04	−0.86
BMI	0.00	−0.00	0.01	0.14	−0.03	−0.68	−0.04	−0.78
SES	0.07	1.44	0.07	1.39	−0.09	−1.90	−0.08	−1.68
T1 measure	0.55	12.33 ***	0.51	10.84 ***	0.49	11.37 ***	0.49	10.59 ***
**Step 2**								
C-T1			0.11	2.34 *			−0.10	−2.24 *
	Total *R*^2^ = 0.33, *F* (6, 358) = 29.68 ***, Δ*R*^2^ = 0.010				Total *R*^2^ = 0.23, *F* (6, 358) = 23.57 ***, Δ*R*^2^ = 0.010			

Note. T1: time 1; T2: time 2; C: conscientiousness; PA: positive affect; NA: negative affect; LS: life satisfaction; PS: physical symptoms; BMI: body-mass index; SES: socioeconomic status. T1 measure corresponds to PA, NA, LS and PS at T1 in each equation, respectively. * *p* < 0.05, ** *p* < 0.01, *** *p* < 0.001.

**Table 4:** Separate regression models for subjective and physical well-being at T1 predicting T2 conscientiousness.

Predictor	C-T2				Predictor	C-T2			
	Model 1		Model 2			Model 1		Model 2	
	β	*t*	β	*t*		β	*t*	β	*t*
**Step 1**					**Step 1**				
Gender	−0.15	−1.61	−0.14	−1.48	Gender	−0.15	−1.61	−0.15	−1.60
Age	0.02	0.49	0.02	0.43	Age	0.02	0.49	0.02	0.51
BMI	−0.03	−0.76	−0.03	−0.77	BMI	−0.03	−0.76	−0.03	−0.73
SES	0.07	1.76	0.07	1.68	SES	0.07	1.76	0.07	1.75
C-T1	0.65	16.39 ***	0.64	15.00 ***	C-T1	0.65	16.39 ***	0.65	15.99 ***
**Step 2**					**Step 2**				
PA-T1			0.03	0.69	NA-T1			−0.01	−0.14
	Total *R*^2^ = 0.44, *F* (6, 358) = 47.40 ***, Δ*R*^2^ = 0.001					Total *R*^2^ = 0.44, *F* (6, 358) = 47.26 ***, Δ*R*^2^ = 0.000			
	**C-T2**					**C-T2**			
	**Model 1**		**Model 2**			**Model 1**		**Model 2**	
	**β**	***t***	**β**	***t***		**β**	***t***	**β**	***t***
**Step 1**					**Step 1**				
Gender	−0.15	−1.61	−0.15	−1.61	Gender	−0.15	−1.61	−0.15	−1.60
Age	0.02	0.49	0.02	0.49	Age	0.02	0.49	0.02	0.49
BMI	−0.03	−0.76	−0.03	−0.80	BMI	−0.03	−0.76	−0.03	−0.75
SES	0.07	1.76	0.06	1.42	SES	0.07	1.76	0.07	1.75
C-T1	0.65	16.39 ***	0.63	14.89 ***	C-T1	0.65	16.39 ***	0.65	15.87 ***
**Step 2**					**Step 2**				
LS-T1			0.07	1.51	PS-T1			−0.01	−0.18
	Total *R*^2^ = 0.45, *F* (6, 358) = 47.94 ***, Δ*R*^2^ = 0.004					Total *R*^2^ = 0.44, *F* (6, 358) = 47.27 ***, Δ*R*^2^ = 0.000			

Note. T1: time 1; T2: time 2; C: conscientiousness; PA: positive affect; NA: negative affect; LS: life satisfaction; PS: physical symptoms; BMI: body-mass index; SES: socioeconomic status. T1 measure corresponds to PA, NA, LS and PS at T1 in each equation, respectively. *** *p* < 0.001.

## Data Availability

The data presented in this study are available upon reasonable request from the corresponding author.
